# Female Community Health Volunteers in Community-Based Health Programs of Nepal: Future Perspective

**DOI:** 10.3389/fpubh.2017.00181

**Published:** 2017-07-21

**Authors:** Resham Bahadur Khatri, Shiva Raj Mishra, Vishnu Khanal

**Affiliations:** ^1^Center for Research and Development, Kathmandu, Nepal; ^2^School of Public Health, The University of Queensland, Brisbane, QLD, Australia; ^3^School of Public Health, Curtin University, Perth, WA, Australia

**Keywords:** community-based, female community health volunteers, maternal and child health, Nepal, roles

## Abstract

Nepal’s Female Community Health Volunteers (FCHVs) program started in 1988. In the early years of program initiation, FCHVs were assigned to promote and distribute the family planning commodities such as condoms and pills. Over past three decades, FCHVs’ roles have gradually expanded beyond family planning program and especially are focused on maternal and child health services at a large scale. FCHVs are an integral part of many community-based health programs, and their roles are instrumental in linking families and communities to community health workers and periphery-level health facilities. However, the fragmented nature of health programs poses a challenge for these health volunteers to coordinate activities and deliver the results. This perspective aims to review their contribution, challenges and recommend an integrated FCHV program model to support in the implementation of the community-based health interventions effectively.

## Introduction

Female Community Health Volunteer (FCHV) program started in 1988. In the early years of program initiation, married women of reproductive age were selected as FCHVs and assigned to promote and distribute the birth-spacing commodities such as condoms and pills, with the sole purpose of supporting family planning program in Nepal. After almost three decades, these health volunteers have become an indispensable part of community-based health programs in Nepal (1, 2). Currently, over 52,000 FCHVs are actively working—roughly one in 500 people or at least one in each ward of Village Development Committee, the smallest local administrative body, as non-paid community health cadres (3). The current FCHV guideline has provisioned that the FCHVs must be married and reside in the ward where they are expected to serve. Mothers’ group for health (a group of women active in different local social and health activities within their locality) selects them to work within their community. After selection, they get 18 days of basic training on family planning, maternal, newborns, child health, and nutrition issues (4). Earlier, the FCHVs were assigned to act as health promoters and dispensers of health commodities, but later with few exceptions, they are also serving as health service providers, notably treating childhood pneumonia and diarrhea at the community (5–7).

## Roles of FCHVs in Community-Based Health Programs

Female Community Health Volunteers are playing important roles in the implementation of many community-based maternal and child health programs such as National Immunization Program, Birth Preparedness Package, Community-Based Integrated Management of Neonatal and Childhood Illness (CB-IMNCI), Integrated Management of Acute Malnutrition, Infant and Young Child Feeding, and Family Planning program (3). In these programs, FCHVs are expected to accomplish several health activities in the community. Categorically, these activities can be divided into health promotion and dispensing of community health commodities, and treatment and referral services (6). For health promotion and dispensing works, they conduct mothers’ group meetings, carry out counseling sessions on birth preparedness and complication readiness, distribute iron folate tablets to pregnant women, and distribute family planning commodities such as pills and condoms. They have also been mobilized to support the national campaigns like the national polio campaign and vitamin A capsule distribution to under-five children (4). As far as the treatment and referral role is concerned, FCHVs identify the childhood pneumonia and provide oral antibiotic (cotrimoxazole) if needed. In the event of childhood diarrhea, they provide zinc tablets and oral rehydration solution. Likewise, they also identify the danger signs in postpartum women and newborns during postnatal home visits and advise the new mothers for providing thermal care to the babies with low birth weight (8). The community health interventions which are implemented with the support of FCHVs, have had high population-level coverage, for instance, NDHS 2001, 2006, and 2011 (9–11) revealed that many interventions that are implemented through FCHVs had had in increasing trend and consistent population-level coverage (Table 1).

**Table 1:** Female Community Health Volunteers (FCHVs) performance in community-based health programs.

Health promotion and dispensing activities	Number (NDHS 2001)	FCHV’s contribution (%)	Number (NDHS 2006)	FCHV’s contribution (%)	Number (NDHS 2011)	FCHV’s contribution (%)
Pregnant women received counseling on side effects of family planning commodities	2,952	66.4	1,471	55.5	1,533	64.6
Pregnant women received birth preparedness counseling	4,745	47.6	4,066	57.3	4,148	75.5
Pregnant women received Iron tablets in the past 6 months	4,745	22.7	4,066	59.3	4,148	79.5
Children (6–59 months) received vitamin A capsule	6,293	81	4,768	87.5	4,360	90.4
Children (12–59 months) received deworming tablets in the past 6 months	0	0	4,274	81.8	3,853	83.7
**Health services and treatment provided**	**Number [HMIS 2013]**	**FCHV’s contribution (%)**	**Number (HMIS 2014)**	**FCHV’s contribution (%)**	**Number (HMIS 2015)**	**FCHV’s contribution (%)**
Children aged under 5 years with diarrhea treated with oral rehydration solution and zinc	1,753,758	62	1,766,903	64	1,413,111	64
Children aged under 5 years with pneumonia treated with cotrimoxazole	2,827,759	57	2,671,922	58	2,208,221	57

The remarkable progress on these indicators is mainly contributed by FCHVs who played pivotal roles in the dissemination of health information, and distribution of health commodities at the community level. Similarly, the annual health report based on Health Management Information System (HMIS) 2015 also reported that FCHVs treated about three of five childhood (aged under 5 years) diarrhea and pneumonia (12) (Table 1). However, the revised CB-IMNCI protocol 2014 (13) states that FCHVs are no longer eligible to treat childhood pneumonia in the community. This revision is supposed to affect the treatment of childhood pneumonia among many hard-to-reach, remote, and disadvantaged communities, where pneumonia is still a major cause of under-five deaths (13).

## Challenges of FCHVs Program

Although the FCHVs have been making significant contributions to the health system of Nepal, this program is facing several challenges at the policy and implementation level. At the policy level, major challenges are about the ownership and verticalization of community components of health programs (14). The Family Health Division is responsible for the maternal health programs, for instance, Safe Motherhood Program and Birth Preparedness Package. This division is also a focal point for FCHV program, where an officer is positioned to conduct FCHVs annual review meetings and maintain their profile (15). Similarly, the Child Health Division looks after Immunization, Nutrition, and CB-IMNCI programs (16). The major bulk of the FCHV’s activities such as biannual supplementation of vitamin A and deworming tablets to under-five children, iron tablets to pregnant women, actions against undernutrition, and other nutritional promotional campaigns at the community lies in the domain of Nutrition and CB-IMNCI programs (17). Most of the maternal and child health programs have separate community components for FCHVs. Furthermore, duplications are observed in the community activities between and within the programs of health divisions (18). For instance, neonatal health issues are included in the Child Health Division’s CB-IMNCI program and Family Health Division’s safe motherhood program. Likewise, nutritional contents such as treatment of malnutrition of under-five children and nutrition promotion are included in Child Health Division’s CB-IMNCI and Nutrition programs (15). These kinds of duplications create the situation of vertical planning and management for the same program or similar purpose.

Also, because of programmatic fragmentations, FCHVs are found to be overburdened. As a result, they are obliged to perform multiple tasks for the similar activities listed in different programs (14). They are needed to train with separate action cards, job aids, treatment protocols, counseling guidelines and so forth for each community health program separately. In many situations, FCHVs become confused on different recording and reporting tools, and they face difficulties in filling up these forms (14, 16). It is to be noted that these volunteers are not highly educated and therefore putting a lot of recording and reporting burden on them might be detrimental to their volunteering actions (14, 19). Sometimes, these volunteers are also being used in other non-health programs such as forest users group, community development groups, education and microcredit and saving groups (14). It means that they have to spend more hours in other volunteer works beyond health sectors. Not surprisingly, therefore, there is a declining sense of motivation among the FCHVs being coupled with minimal event based incentives offered to them for their volunteer’s support (20).

## Integrated FCHV Program

The Government of Nepal has acknowledged that the FCHVs have contributed significantly to achieve the milestones of the Nepal’s Millennium Development Goals 4 and 5 by providing basic health services to women and children in the community. However, in the Sustainable Development Goal-era, their roles need some contextual modifications. They should be promoted as a bridge between families and communities to periphery-level health facilities but not as servicer providers with the exception in some remote areas. They can counsel mothers, dispense health commodities such as zinc, ORS, pills, or condoms, and refer women and children to the appropriate health workers or health facilities when needed (3).

Moreover, it is necessary to revitalize the FCHVs training curricula included in different community-based health programs. Revitalization can be materialized with two approaches: first, the structural reform in the FCHVs program and second the integration of training curricula and programmatic contents into a single package. The organizational or structural reform could be the establishment of FCHVs unit including provision needed human resources at the various level of health organogram. Such unit would be responsible for planning and implementation of FCHVs based health interventions. And the integrated FCHV training curricula will help to reduce the work burden and harmonize the community health interventions. So, an integrated FCHV program can be designed in a continuum of care model—bringing all the prenatal, perinatal, and postnatal and child health training contents under a single umbrella. Such integrated program could also link the fragmented activities of community-based health programs in a continuum of care model (8) (Table 2).

**Table 2:** Contents of proposed integrated Female Community Health Volunteer program.

Prepregnancy module	Prenatal module	Perinatal module	Postnatal module	Childhood module	Cross-cutting module
Educating women of reproductive age women on family planning Counseling on preconception and maternal nutrition during prepregnancy Counseling on hygiene and sanitation practices	Educating pregnant women for promotion of birth preparedness and complication readiness Promotion of ANC care, tetanus toxoid, and institutional delivery Promotion of logo iodized salt Dietary diversification and promotion of diversified food during pregnancy Counseling on promotion of maternal micronutrient during pregnancy Counseling and distribution of iron foliate, calcium, chlorhexidine gel, misoprostol tablet	Promotion of clean and safe delivery practices Promotion of hand washing and sanitation activities Educating mothers on essential newborn care Educating mothers on risk of low birth weight babies, advice for skin to skin contact Counseling on complication of non-breathing babies at birth Counseling on exclusive breastfeeding Counseling and distribution of vitamin A, misoprostol for prevention postpartum hemorrhage in case of home delivery, chlorhexidine gel, iron folate tablets for postpartum period	Educating mothers on dangers signs of postpartum mothers and newborn and referral if needed Counseling on postpartum family planning methods Counseling on infant and young child feeding and complementary feeding practices Promotion of child growth monitoring and childhood immunization Nutrition education on micronutrient, dietary diversification, and fortified food vitamin A, iron folate to mothers	Counseling and identification of dangers signs of common childhood illness (pneumonia, diarrhea, acute jaundice malnutrition) and referral if needed Counseling on home-based management of childhood diarrhea and pneumonia Counseling and promotion of childhood nutrition interventions Counseling and promotion of child immunization Distribution of vitamin A, iron tablets, oral rehydration packet, zinc tablets to children Promotion and distribution of condoms and pills for eligible couple at the community	Providing counseling on prevention/protection of other communicable disease like HIV/AIDS Support other behavior change communication campaigns and social activities Providing home health education and advice on diet and lifestyle Educating primary level school students on making healthy tiffin and promoting healthy canteen Providing information on non-communicable disease services available at the health facilities

In addition, given the changing epidemiological milieu in the country where non-communicable diseases (NCDs) are currently the leading cause of mortality and morbidity, they should be given some roles in disseminating health promotion messages on diet and lifestyles. Such integrated program including some roles on home-based health education for NCDs could mean an end to many vertical programs (Table 2).

## Conclusion

Female Community Health Volunteers are frontline pillars of community-based health programs in Nepal. They have been successful in making a significant contribution to various community-based maternal and child programs in various capacities as health promoters, dispensers, and service providers. However, the fragmented roles of FCHV program need to be reorganized comprehensively, structurally, and programmatically. It is crucial for the health authorities to craft an integrated FCHV program model and remove/merge the duplicated roles in various community-based maternal and child health programs. An integrated FCHV program can be cost effective community-based program package, which can increase FCHVs’ productivity.

## Author Contributions

RK designed concept, review literature and drafted the paper. SM and VK provided support in revision. All the authors read the final version of the papers and approved it for publication.

## Conflict of Interest Statement

The authors declare that the research was conducted in the absence of any commercial or financial relationships that could be construed as a potential conflict of interest.
